# An Unusual Location for a Nonurachal Bladder Adenocarcinoma

**DOI:** 10.1155/2021/5827120

**Published:** 2021-09-23

**Authors:** George Khludenev, Akshay Reddy, Sinan Akosman, Michael J. Whalen

**Affiliations:** George Washington University School of Medicine, 2300 I St NW, Washington, DC 20052, USA

## Abstract

Malignant bladder neoplasms represent a significant disease burden not only for urologists but also the broader medical community. While the majority of bladder tumors are urothelial in origin, up to two percent are found to be adenocarcinomas. Among bladder adenocarcinomas, roughly one-tenth are urachal and are frequently located at the dome of the bladder where urachal remnants can often be found. We describe a case of bladder adenocarcinoma that presented at the dome of the bladder but ultimately exhibited a nonurachal histology. A 65-year-old male with a history of myocardial infarction and cerebrovascular accident with residual right-sided hemiparesis and aphasia was referred to our clinic for evaluation of a bladder mass discovered in the setting of painless gross hematuria. Diagnostic cystoscopy demonstrated a large mass at the dome of the bladder, and subsequent transurethral resection revealed stage T1 mucinous adenocarcinoma arising in a villous adenomatous lesion without the presence of muscle in the specimen. The patient underwent a robotic-assisted laparoscopic partial cystectomy with extended bilateral pelvic lymph node dissection. Postoperatively, the patient experienced short-lived paralytic ileus and was discharged on postoperative day 5. Follow-up surveillance imaging at 6 months with CT chest, abdomen, and pelvis, repeat office cystoscopy, and negative tumor markers postoperatively indicated no evidence of disease recurrence. Characterization of bladder adenocarcinomas into urachal and nonurachal subtypes is critical in differentiating the operative management and oncologic outcomes of the respective neoplasms. However, given the paucity of literature describing treatment approaches to bladder adenocarcinoma in general, existing methods have largely mirrored genetically similar neoplasms, including ovarian and colon adenocarcinomas. Although there is still much to be understood regarding the potential mechanisms of carcinogenesis of nonurachal adenocarcinomas, further investigation may pave the way for a more standardized treatment paradigm and provide insight into the potential utility of modern immunotherapies.

## 1. Introduction

With an overall lifetime risk of approximately 2.2% in the general population, bladder cancer represents the sixth most common cancer in the United States and is expected to affect one out of every 27 men and one out of every 89 women during their lifetime [1, 2]. While most bladder tumors demonstrate urothelial histology, adenocarcinoma accounts for only 0.5-2% of cases and typically exhibits invasive behavior. Adenocarcinomas of the bladder are further classified based on their developmental origin from the urachus, an embryological remnant of the fibrous allantois that connects the developing bladder to the umbilicus [3]. Since the allantois connects the umbilicus to the dome of the bladder, urachal adenocarcinomas are often found in this location. They are actually far less common than their nonurachal counterparts, only accounting for an estimated 10% of bladder adenocarcinomas [2]. Nonetheless, given the rarity of adenocarcinoma of the bladder (0.5-2% in the United States) compared to other, more prevalent histological subtypes such as urothelial or squamous cell, there is a paucity of data and literature discussing either urachal or nonurachal bladder malignancies [3]. Here, we describe the presentation and management of a patient with nonurachal, primary bladder adenocarcinoma at the dome of the bladder.

## 2. Case Presentation

A 65-year-old male was referred to our clinic for evaluation of a bladder mass discovered in the setting of painless gross hematuria. The patient's past medical history was notable for a myocardial infarction 4 years prior, and a cerebrovascular accident 3 years prior with residual right-sided hemiparesis and aphasia, rendering him wheelchair bound.

**Figure 1:**
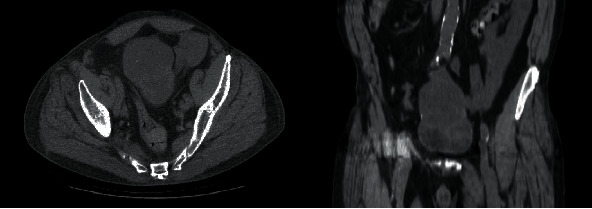
Axial and coronal cross-sectional imaging via CT abdomen and pelvis revealing an exophytic mass arising at dome of bladder.

**Figure 2:**
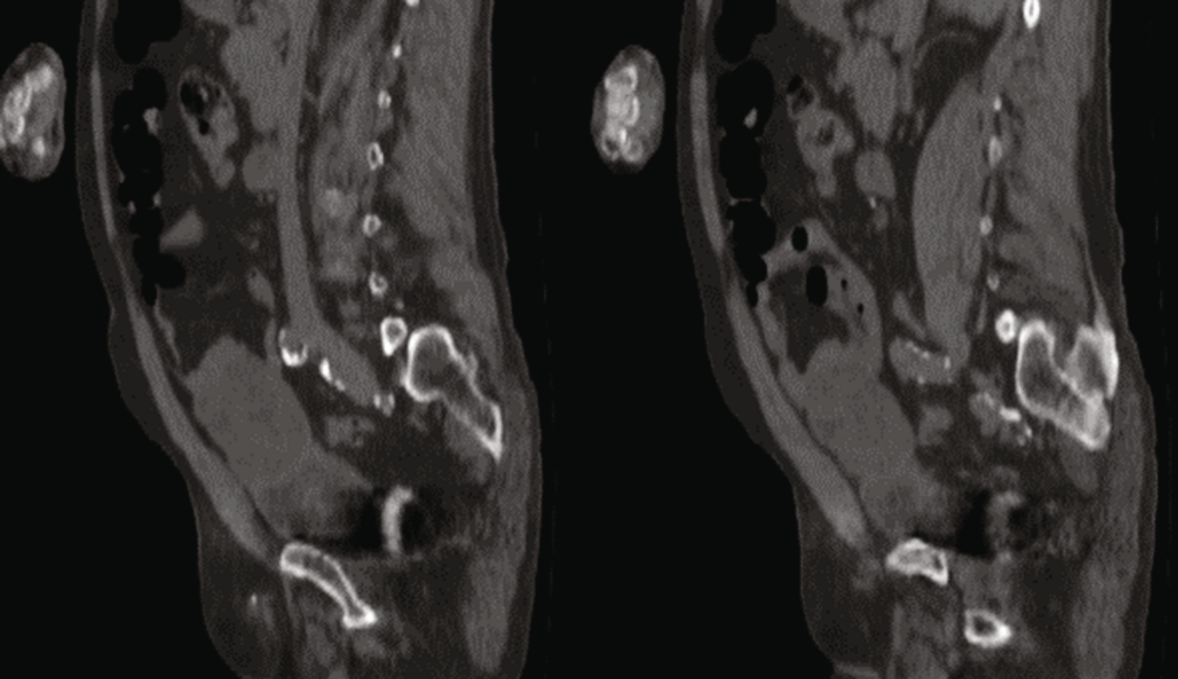
Sagittal cross-sectional imaging via CT abdomen and pelvis revealing an exophytic mass arising at the dome of the bladder.

Cross-sectional imaging via CT abdomen and pelvis revealed an exophytic mass at the bladder dome measuring 7.8 × 5.9 cm with central necrosis and a thick, enhancing wall as well as probable communication with the lumen of the urinary bladder (Figures 1 and 2). There was no hydronephrosis or pelvic lymphadenopathy. Metastatic survey was negative. Diagnostic cystoscopy demonstrated a large mass at the dome of the bladder, and subsequent transurethral resection revealed stage T1 mucinous adenocarcinoma arising in a villous adenomatous lesion without the presence of muscle in the specimen. Serum tumor markers revealed elevated CEA at 131 ng/mL (ULN 4.7 ng/mL).

**Figure 3:**
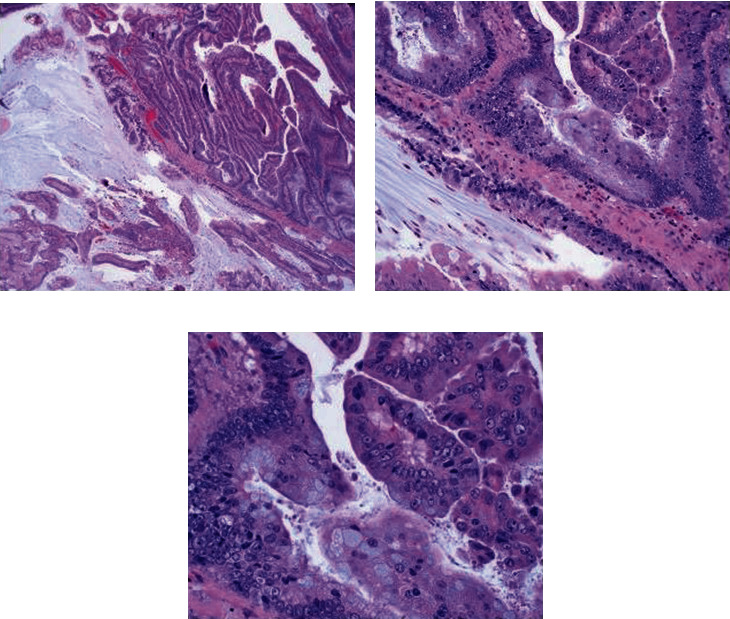
Representative pathology at (a) 4x and (b) 20x demonstrating villous architecture arising from mucinous adenocarcinoma and (c) 40x with presence of mucin producing goblet cells.

**Figure 4:**
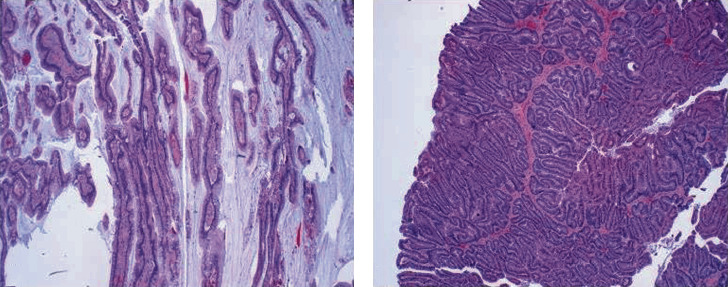
(a) Villous architecture 4x. (b) Glandular architecture of nonurachal adenocarcinoma 4x.

After thorough counseling and informed consent about the potential role of perioperative chemotherapy the patient elected for surgical management and underwent a robotic-assisted laparoscopic partial cystectomy with extended bilateral pelvic lymph node dissection. Although no muscle-invasion had been seen on TURBT pathology, the advanced radiographic appearance of the tumor, with concern for extravesical extension, justified the surgical approach. Intraoperatively, no gross metastatic lesions in the abdomen were seen and the tumor was resected with 1.5 cm margins. All twenty-four dissected lymph nodes were negative for carcinoma. Final pathology revealed pT2b with tumor involvement of the surface urothelium and invasion of the deep muscularis propria. Pathologic evaluation further revealed that, although the location of this tumor at the dome of the bladder suggested urachal adenocarcinoma as the final diagnosis, no urachal glandular remnants were identified and so the features were most strongly suggestive of a primary nonurachal bladder adenocarcinoma (Figures 3 and 4).

Postoperatively, the patient experienced paralytic ileus but otherwise did well and was discharged on postoperative day 5. He was counseled about the role of adjuvant chemotherapy but elected to defer given the lack of robust evidence. Follow-up surveillance imaging at 6 months with CT chest, abdomen, and pelvis revealed no evidence of disease recurrence. He also underwent a normal office cystoscopy and had negative tumor markers, indicating no evidence of disease. The patient remained disease free at 24-month follow-up.

Molecular profiling of the tumor using the Foundation One CDx test reported microsatellite stability and a tumor mutational burden of 4 mutations per megabase. The test also described four genomic alterations: G12D on the KRAS gene, R201C on the GNAS gene, Q604 on the RMB10 gene, and R248W on TP53 gene. Targeted therapeutic options for this tumor type are limited; however, treatments targeting similar tumor subtypes have shown promise.

## 3. Discussion

Beyond the origin of disease, urachal and nonurachal adenocarcinoma can also be differentiated by their presentation, management, and oncologic outcomes (Table 1). The nonurachal subtype is more common in younger patients (median 69 yo vs. 56 yo) and, historically, has been noted to present later in the disease course with metastatic or lymph node spread [2, 4]. Although bladder dome involvement was noted in the pathology of our patient with nonurachal adenocarcinoma, it is important to note that the anterior wall and dome are most often associated with urachal tumors [2, 4]. The etiology of bladder adenocarcinomas is not entirely well established but is hypothesized to be a result of a metaplastic transformation of the cells within von Brunn's nests to cystitis glandularis/cystica and later to adenocarcinoma, a sequence triggered by chronic inflammation [5]. Histologically, urachal tumors are classified broadly into two categories: mucinous cystic and noncystic adenocarcinomas. Primary adenocarcinomas are similar in nature to noncystic adenocarcinomas and are classified enteric-type, mucinous (with or without signet ring cells) and not otherwise specified [4]. Specific variants may resemble salient features similar to colon or ovarian adenocarcinoma such as signet ring carcinoma or those originating from villous adenomas, as seen in our patient [5]. From a purely histological standpoint, these bladder neoplasms do differ from colonic and ovarian adenocarcinoma in staining patterns of B-catenin (membranous in bladder, nuclear in colonic), positivity of keratin 7 (more common in bladder), and keratin 20 (more common in colonic) and usually negative for OC125 (typically positive in ovarian/cervical glandular tumors) [5]. Genetically, however, both urachal and nonurachal subtypes tend to express molecular markers more similar to colorectal or ovarian adenocarcinomas, rather than urothelial bladder tumors. Both glandular neoplasms of the bladder and colon are more likely to express mutations in the TP53, APC (Wnt pathway), and KRAS [6, 7]. The genetic profiling data from our patient's tumor is consistent with what has been described in the existing literature. Broadly characterized for bladder neoplasms in general, TP53 mutations have been far more thoroughly described (up to 50%) as potential drivers of carcinogenesis [8]. More specifically, though limited by patient sample sizes, the current genetic profiling data of mucinous type primary bladder adenocarcinomas highlight KRAS (MAPK pathway) mutations in up to 67% of tumors [7]. Likewise, the specific G12D mutation in our patient is also a characteristic of glandular neoplasms in gastrointestinal sites, including the pancreas and colon. Though no viable immunotherapies have been determined to specifically target this KRAS mutation, several novel contenders do exist from other primary sites and have potential to be extrapolated to benefit a wider range of solid malignancies, including the bladder. Though preliminary, the use of primed cytotoxic T cell therapies to target KRAS mutations in colorectal carcinoma have shown some oncologic success in targeting neoantigens produced by the mutant G12D tumor cells and presented on HLA class I molecules [9].

As with any rare disease, it is difficult to make conclusive outcome-based statements with a high degree of power. However, when exclusively evaluating survival differences, Luzzago et al. have retrospectively provided the most comprehensive and recent data comparing the survival outcomes of urachal vs. nonurachal subtypes using the Surveillance, Epidemiology and End Results (SEER) database [10]. Despite the fact that it is less common, five-year cancer-specific survival in the nonmetastatic setting was shown to favor urachal versus nonurachal (68 vs. 49%, respectively, HR 0.6; *p* = 0.01) [10]. Though these comparisons were adjusted for potential confounding variables, discrepancies in treatment approach and unmeasured clinicopathologic features may have influenced these findings.

From a treatment perspective, urachal tumors are more likely to be managed with partial cystectomy (73.9 vs. 14.8%), with some older studies indicating up to 83% of patients with urachal tumors treated with partial cystectomy (PC) [10, 11]. There is greater variation in the management of nonurachal tumors, and recently, there has been an increasing interest in the utility of adjuvant chemotherapy and even external beam radiation therapy (EBRT) in the setting of locally advanced disease and/or positive surgical margin. In our patient, partial cystectomy was an appropriate option due to the concern for urachal pathology due to location at the dome, exophytic nature of the tumor, and ability to maintain adequate postoperative functional bladder capacity. In assessing the outcomes of PC vs. RC (radial cystectomy), a recent retrospective study evaluating the SEER database has shown no significant difference in the five-year overall survival in nonmetastatic, T1-2 adenocarcinoma treated with PC vs. RC (PC = 27% and RC = 21%, *P* = 0.9). Of note, such retrospective analysis is subject to treatment selection bias, since more PC patients were stage T1 as opposed to T2 [12].

While the role of adjuvant chemotherapy in bladder urothelial carcinoma has been explored in the literature, there is less robust evidence for the role in adenocarcinomas. A meta-analysis by Leow et al. demonstrated a 23% reduction in relative risk of death (HR 0.77) for patients with muscle-invasive bladder cancer undergoing cisplatin-based adjuvant chemotherapy after radical cystectomy [13]. For primary bladder adenocarcinoma, the situation is more complicated given the lack of standardized chemotherapy regimens. Most of the approach is extrapolated from the colorectal cancer literature for which locally advanced (stage 3 disease) treatment regimens generally consist of FOLFOX (5-FU, leucovorin, and oxaliplatin) or CapeOx (capcitabine and oxaliplatin) and may not be directly applicable [14]. Berg et al. have recently reported no improvement in median survival in patients with nonmetastatic, muscle-invasive adenocarcinoma/urothelial cancer with glandular dedifferentiation (pT2N+M0 or ≥pT3N0/N+M0) who underwent RC with adjuvant chemotherapy compared to those who received RC alone (21.79 months vs. 22.60 months) [15]. Davaro et al. described similar findings while evaluating the benefit of EBRT in patients with nonmetastatic cT2-4 nonurachal adenocarcinoma. Five-year survival significantly (*P* < 0.001) favored those who received cystectomy alone (39.6%) compared to cystectomy and EBRT (26.9%), although this finding was likely a consequence of the higher stage and positive margin status of the cystectomy+EBRT group [16]. Data regarding the role of neoadjuvant chemotherapy is also limited, with one small study consisting of 6 patients (3 with urachal and 3 with nonurachal locally advanced disease) showing modest benefit in downstaging of the primary tumor and preventing micrometastasis with a gemcitabine/cisplatin and S-1 (5-FU alternative) regimen [17]. The role of more modern adjuvant immunotherapies in nonurothelial bladder cancers has also not yet been well established. As mentioned previously, recent genetic and molecular evidence suggests greater similarities between bladder to colon adenocarcinomas rather than urothelial tumors. These findings may be indicative of the utility of more novel immunotherapeutics, similar to those used in adenocarcinoma of the colon, though yet to be extensively studied [7]. For example, current ongoing clinical trials of immunotherapeutic agents nivolumab and ipilimumab in addition to targeted molecular therapy (cabozantinib) for rare genitourinary cancers that have metastasized promise to add valuable information [18].

In summary, adenocarcinoma of the bladder is a relatively uncommon and aggressive urologic malignancy that can be subclassified based on the origin of the disease. Neoplasms of the urachus are far more rare than primary adenocarcinoma. In addition, there is much greater variation in the management of nonurachal tumors, especially regarding the role of perioperative chemotherapy and radiation as adjuvant modalities in treating nonurachal adenocarcinoma. Continued research on this specific subclassification of bladder cancer is needed to further characterize and understand its behavior and the associated clinical outcomes.

## Figures and Tables

**Table 1:** Comparison of urachal vs. nonurachal bladder adenocarcinoma presentation.

	Urachal [3]	Non-urachal [3, 5]
Origin of disease	Urachal remnant	Bladder urothelium
Age	5-6th decade of life	6-7th decade of life
Location of disease	Bladder dome, anterior wall	Trigone, posterior wall, but can arise throughout
Immunohistochemical staining	CK20, CDX2 (+/- CK7, B-catenin, HM cytokeratin)	Variable based on histological phenotype
Possible symptoms	Umbilical discharge/bleeding, hematuria, lower urinary tract symptoms	Hematuria, lower urinary tract symptoms

## Abbreviations

CT: Computed tomography
SEER: Surveillance, Epidemiology and End Results (database)
PC: Partial cystectomy
RC: Radical cystectomy
ERBT: External beam radiation therapy
FOLFOX: 5-FU, leucovorin, and oxaliplatin
CapeOx: Capecitabine and oxaliplatin.
